# General Practitioner Antimicrobial Stewardship Programme Study (GAPS): protocol for a cluster randomised controlled trial

**DOI:** 10.1186/s12875-016-0446-7

**Published:** 2016-04-21

**Authors:** Minyon L. Avent, Malene Plejdrup Hansen, Charles Gilks, Chris Del Mar, Kate Halton, Hanna Sidjabat, Lisa Hall, Annette Dobson, David L. Paterson, Mieke L. van Driel

**Affiliations:** The University of Queensland, School of Public Health, Herston, QLD 4006 Australia; The University of Queensland, UQ Centre for Clinical Research, Herston, QLD 4006 Australia; Centre for Research in Evidence-Based Practice, Faculty of Health Sciences and Medicine, Bond University, Robina, QLD 4226 Australia; Institute of Health and Biomedical Innovation and School of Public Health & Social Work, Queensland University of Technology, Kelvin Grove, QLD 4059 Australia; The University of Queensland, Discipline of General Practice, School of Medicine, Herston, QLD 4006 Australia

**Keywords:** General practice, Primary care, Antimicrobial stewardship, Antibiotics, Antimicrobial resistance, Protocol

## Abstract

**Background:**

There is a strong link between antibiotic consumption and the rate of antibiotic resistance. In Australia, the vast majority of antibiotics are prescribed by general practitioners, and the most common indication is for acute respiratory infections. The aim of this study is to assess if implementing a package of integrated, multifaceted interventions reduces antibiotic prescribing for acute respiratory infections in general practice.

**Methods/design:**

This is a cluster randomised trial comparing two parallel groups of general practitioners in 28 urban general practices in Queensland, Australia: 14 intervention and 14 control practices. The protocol was peer-reviewed by content experts who were nominated by the funding organization.

This study evaluates an integrated, multifaceted evidence-based package of interventions implemented over a six month period. The included interventions, which have previously been demonstrated to be effective at reducing antibiotic prescribing for acute respiratory infections, are: delayed prescribing; patient decision aids; communication training; commitment to a practice prescribing policy for antibiotics; patient information leaflet; and near patient testing with C-reactive protein.

In addition, two sub-studies are nested in the main study: (1) point prevalence estimation carriage of bacterial upper respiratory pathogens in practice staff and asymptomatic patients; (2) feasibility of direct measures of antibiotic resistance by nose/throat swabbing.

The main outcome data are from Australia’s national health insurance scheme, Medicare, which will be accessed after the completion of the intervention phase. They include the number of antibiotic prescriptions and the number of patient visits per general practitioner for periods before and during the intervention. The incidence of antibiotic prescriptions will be modelled using the numbers of patients as the denominator and seasonal and other factors as explanatory variables. Results will compare the change in prescription rates before and during the intervention in the two groups of practices.

Semi-structured interviews will be conducted with the general practitioners and practice staff (practice nurse and/or practice manager) from the intervention practices on conclusion of the intervention phase to assess the feasibility and uptake of the interventions.

An economic evaluation will be conducted to estimate the costs of implementing the package, and its cost-effectiveness in terms of cost per unit reduction in prescribing.

**Discussion:**

The results on the effectiveness, cost-effectiveness, acceptability and feasibility of this package of interventions will inform the policy for any national implementation.

**Trial registration:**

The GAPS trial is registered under the Australian New Zealand Clinical Trials Register, reference number: ACTRN12615001128583 (registered 26/10/2015).

## Background

Australia is one of the highest consumers of antibiotics in the developed world with 45 % of the Australian population being supplied at least one antibiotic per year [1]. The defined daily dose (DDD) in Australia is nearly 23/1000 population/day [1] compared with about 18 DDD/1000 population /day in Denmark and less than 11 DDD/1000 population /day in general practice in the Netherlands [2–4].

There is a strong link between antibiotic consumption and the rate of antibiotic resistance [5]. Acute respiratory tract infection (ARIs) are the most common reason for prescribing an antibiotic in primary care [6]. In Australia antibiotic resistance in common pathogens causing ARIs has increased over the past 20 years [7]. For example, resistance of *Streptococcus pneumoniae* to macrolide antibiotics has increased from 8.7 % in 1994 to 20.4 % in 2007, and this trend is continuing [8]. Patients with infections caused by antibiotic-resistant organisms have an increased mortality compared with those infected with antibiotic-susceptible organisms [9, 10].

General Practitioners (GPs) have the potential to be the most influential health care professionals to address the problem of antibiotic resistance as the majority of antibiotics are prescribed in the general practice setting and antibiotics remain the most common class of medicine prescribed [11]. Continued improvements in prescribing practice and a positive influence on individual and community beliefs about antibiotic consumption are essential to limit the spread of antibiotic resistance [12]. Antibiotics are often inappropriately prescribed for patients with ARIs [6]. Research shows that up to half of antimicrobials prescribed in Australian hospitals are discordant with guidelines or microbiological results and hence are considered inappropriate,[13] however, little is known about what happens in the primary care setting [14].

Unfortunately, new antimicrobials are not being developed at a pace that comes anywhere close to meeting the urgent need; therefore, the healthcare system needs to undertake efforts that save one of medicine’s most precious and long-standing resources [15]. This was summarised by the World Health Day 2011 slogan ‘Combat antibiotic resistance: no action today, no cure tomorrow’. Reducing the inappropriate use of antimicrobials has been shown to improve patient outcomes and reduce adverse consequences of antibiotic use (including antibiotic resistance, toxicity and unnecessary costs) [16].

Antimicrobial stewardship (AMS) is the coordinated actions designed to promote and increase the appropriate use of antimicrobials and is a key strategy to conserve the effectiveness of antibiotics. Australia’s first National Antimicrobial Resistance strategy for 2015–2019 states that there is a need for resources to support the implementation of AMS for all settings including primary health care [17].

There are a number of interventions that have shown promise at decreasing antibiotic prescribing for ARIs in primary care: delayed prescribing; patient decision aids; communication training; near patient testing with C-reactive protein and commitment to a practice prescribing policy for antibiotics [18–20]. Prescribers are well placed to convey the importance of informing patients that they are twice as likely to carry resistant bacteria after a course of antibiotics as someone who has not taken them [21–23]. Evidence from general practice demonstrates that patient satisfaction is linked more with good communication than a prescription for an antibiotic [24, 25]. Several studies have demonstrated that GPs trained in communication skills, [26, 27] and specifically in Shared Decision Making [28–30], prescribed antibiotics significantly less than GPs without training. The benefits of patients managed by a GP trained in enhanced communication skills can persist for at least 3 years, and do not appear to compromise repeat consultation rate, patient recovery or patient satisfaction [26, 27, 31, 32].

Many of these strategies have not been adopted in Australia so there is no evidence about their efficacy in this context, and all have been evaluated in isolation. Evidence from other areas of healthcare suggests that using multiple strategies or interventions in concert could have an even greater impact on prescribing behaviour and induce longer term behaviour change. This could enable clinicians and health care systems to reduce antimicrobial resistance in the future [33].

### Aim

The aim of our study is to assess if implementing an integrated, multifaceted package of interventions reduces antibiotic prescribing for ARIs in general practice.

### Sub-studies nested within the main study

Two specific studies are nested in the main study:

1. point prevalence estimation carriage of bacterial upper respiratory pathogens in practice staff and asymptomatic patients
2. feasibility of direct measures of antibiotic resistance by nose/throat swabbing.

### Primary objective

Our primary objective is to assess the effectiveness, of an integrated, multifaceted package of interventions for ARIs in general practice.

### Secondary objectives are

1. to assess the feasibility and uptake of the integrated package of interventions for ARIs.
2. to assess the likely costs and cost-effectiveness of implementing the integrated package of interventions for ARIs.
3. to estimate the prevalence of bacterial upper respiratory pathogens in asymptomatic general practice staff and patients
4. to assess the feasibility of direct measures of antibiotic resistance by nose/throat swabbing.

## Methods

The trial protocol was developed by researchers at the University of Queensland, Bond University and Queensland University of Technology in Australia in accordance with the CONSORT statement extension to cluster randomised trials [34].

### Study design

This is a clustered randomised parallel group controlled trial.

### Study setting

This study is being conducted in South East Queensland, Australia. Twenty-eight urban general practices have been purposely recruited and randomised to either the control or intervention group.

### Eligibility criteria

All GPs from the recruited general practices were eligible to participate in the study provided they gave consent for the research team to obtain their data on antibiotic prescribing and patient visits from Medicare. General practice staff and patients attending the GP practice for consultation with non-infectious complaints were eligible for the point prevalence nose and throat swab study: asymptomatic carriage of bacterial upper respiratory pathogens.

### Implementation of interventions

An integrated, multifaceted package of interventions will be implemented in the intervention practices by research coordinators who have been trained in the use of the interventions. The GPs in the control practices will continue normal clinical practice while the GPs in the intervention practices will be trained in the interventions as described below. In the six month study period, the research coordinators will regularly visit the intervention practices to support uptake of the interventions and provide any necessary supplementary training.

### Interventions

Evidence based interventions already demonstrated to be effective at reducing antibiotic prescribing for ARIs elsewhere in the world were selected [33]. They were combined into an integrated, multifaceted package with the following components:

1. *Poster on Practice Antibiotic Prescribing Policy*
  This intervention consists of displaying a poster-sized prescribing policy in the GPs waiting room and/or examination room. GPs are encouraged to insert their photograph as endorsement on the poster. The poster, written at the eighth grade reading level in English emphasises the GPs’ commitment to guidelines, i.e. *Therapeutic Guidelines: Antibiotic*, [35] for appropriate antibiotic prescribing and explains why antibiotics are not appropriate in many cases [18].
2. *Patient information leaflet*
  The leaflet provides information to the patient about inappropriate use of antibiotics for ARIs and the potential harmful effects of antibiotics. It complements the poster in the GPs waiting room and/or examination room.
3. *Online communication training package*
  An online communication module is offered in combination with background information on the problem of antimicrobial resistance in primary care and the effectiveness of antibiotics for most commonly presenting ARIs. The module is based on the GRACE INTRO study [19] and has been adapted carefully for the Australian context as part of the Changing the Antibiotic Prescribing of General Practice (ChAP study) (https://www.anzctr.org.au/Trial/Registration/TrialReview.aspx?id=366836&isReview=true), a controlled trial funded by Therapeutic Guidelines Ltd (http://www.tg.org.au/index.php?sectionid=505).
  The online communication training is targeted at GPs rather than patients, and is developed to be sensitive to cultural and national differences. The training in enhanced communication skills focuses on exploring patients’ concerns and expectations, providing information on symptoms, natural course of the disease, treatments, agreement of a management plan, summing up, and providing guidance about when to re-consult. GPs are also provided with a booklet [36] for use during consultations that includes information on symptoms, use of antibiotics that are concordant with *Therapeutic Guidelines: Antibiotic* [35] and antibiotic resistance, self-help measures, and when to re-consult. The training is supported by video demonstrations of consultation techniques and is offered as a Continuing Professional Development activity to GPs.
4. *Delayed antibiotic prescribing*
  The GP can offer the patient a delayed antibiotic prescription. This consists of advice to the patient to only fill the prescription at a pharmacy after a few days if symptoms are not starting to settle or become more severe [37]. A sticker is made available to GPs to apply to the prescription, labelling it as a delayed prescription.
5. *Patient Decision Aids*
  A brief graphical laminated summary of evidence for the management of a number of ARI conditions is provided as a decision aid for use during the consultation. These decision aids have been developed to assist GP and patient to make an appropriate decision about the management of the condition. The Patient Decision Aids support the following conditions:
  acute sore throat;
  acute rhinosinusitis
  acute otitis media; and
  acute bronchitis
6. *Near patient testing: CRP study*
  The CRP test is widely used in some European primary care settings [38] and has been shown to significantly reduce antibiotic prescribing for patients with ARIs [39].
  The intervention practices will each have access to a CRP testing machine for three months (with 50 CRP tests per practice provided free of charge) to determine the feasibility and uptake of this type of near patient testing.
  Tests will be performed using the QuikRead CRP kits (Orion Diagnostica). The research co-ordinator, in conjunction with the distribution company (ABACUS ALS), will train the GPs and practice staff in the use and interpretation of the tests. In addition GPs will have access to an online training module on CRP testing (http://gaps.uq.edu.au).
  The following instructions will be provided regarding CRP testing:
  CRP testing should only be used within ARI consultations for lower respiratory tract infections and acute rhinosinusitis.
  The GP can decide to perform a CRP test as a complement to the routine consultation (including history and physical examination).
  The CRP test is performed on a finger prick blood sample and the result will be available within a few minutes.
  The CRP test result can be used in addition to the clinical assessment to decide whether to prescribe an antibiotic.

### Sample size

The sample size calculation for this study was based on the average change in antibiotic prescription rates in practices in the intervention group (before – after the intervention) compared to the average change in practices in the control group over the same period. For example, an average change in antibiotic prescription rate from 40 to 20 % in the intervention practices and no change in the control practices would result in a difference of 0.2 between the two groups. A difference in average change in rates in the range 0.20–0.25, if the standard deviation in rates was about 0.2, was considered clinically significant and plausible. With equal numbers of practices in the two groups, power of 80 %, significance level of 5 % for a two tailed test, for a difference of 0.24, 12 practices per group would be needed. In fact 14 practices per group were recruited so that a difference of 0.22 would be detectable.

### Recruitment

General practitioners who have consented to participate in the study have been recruited from the selected general practices. General practice staff and patients attending the recruited GP practices for consultation with non-infectious complaints and who have consented to the point prevalence study of asymptomatic carriage of bacterial upper respiratory pathogens have also been recruited.

### Randomisation

Practices were randomly assigned to either the intervention or control arm in a 1:1 ratio. A blocked randomisation list with 8 practices per block was generated using the online software package Sealed Envelope Ltd. 2015 available from: https://www.sealedenvelope.com/simple-randomiser/v1.

### Data collection

In Australia the universal health insurance scheme, Medicare, provides access to medical and hospital services for all Australian residents and some visitors. It includes the Medical Benefits Scheme (MBS) which subsidises the costs of all visits to GPs and medical specialists in non-hospital settings, and the Pharmaceutical Benefits Scheme (PBS) which covers almost all medicines. MBS data include individual records for every patient encounter with a GP; GPs are identified by individual provider codes. PBS data include individual records of every prescription dispensed - from July 2012 this covers all prescriptions, regardless of government subsidies. The prescribing GP is identified by an individual prescriber number. There are legislative constraints on linking MBS and PBS records, but de-identified records can be obtained from the Department of Human Services. For all consenting GPs (in the intervention and control practices) provider records will be obtained for each patient encounter billed to the MBS and prescriber records for each prescription reported to the Pharmaceutical Benefits Scheme (PBS; from July 2012 this covers all prescriptions, regardless of government subsidies). Data will be requested from July 2012 to the end of the intervention period. The data will be extracted a month after the conclusion of the intervention phase in order to ensure that all records have been submitted Department of Human Services. Each prescription will be coded using the Anatomical Therapeutic Chemical (ATC) and those coded J01 (antibacterial for systemic use) are the outcomes of primary interest. Prescription rates will be estimated from the number of antibiotic prescriptions per GP in a specific period (e.g. day or week) and the number of patient encounters for that period and GP as the off-set (i.e., a measure of activity or ‘exposure’).

The cost of the package will be estimated from the perspective of the public health system at the conclusion of the intervention phase of the study. All resources used in implementing the package, including both consumable items and time for all personnel involved, will be identified. Allocation of GP time and practice specific items will be estimated based on responses to questions in the semi-structured interviews. Data on consumables and all centralised staffing will be measured retrospectively by the project co-ordinator using a pre-developed costing spreadsheet. Resources invested in any materials already developed will be considered sunk costs and not included in the cost of the intervention. Resources used solely for research and evaluation purposes will also not be included. Resources will be valued in 2016 Australian dollars using local market prices and labour costs to give a total cost of implementation.

We will also estimate the economic value of the change in antimicrobial usage that results from the package using PBS list prices. This will be combined with our data on the cost of implementing the package to provide a measure of the total changes in costs achieved.

### Data analysis

Prescription rates will be estimated using generalised linear models (e.g., Poisson regression) for the number of antibiotic prescriptions per GP in a specific period (e.g., day or week) with the number of patient encounters for that period and GP as the off-set (i.e., a measure of activity or ‘exposure’). The explanatory variables will be included in the model to account for: intervention vs control practices; periods before and during the intervention; and temporal variables to account for secular trends and seasonal effects. The models will be multi-level to account for nesting of patients within GPs and GPs within practices.

Cost-effectiveness of the package will be estimated from the perspective of the public healthcare system. Results will be presented as the net cost of implementation and as the cost per unit reduction in prescription rates. It is unclear what Australian decision makers are prepared to pay for a unit reduction in prescribing so results will be compared to cost-effectiveness estimates published in the international literature to gauge the efficiency of this package relative to other approaches used to reduce GP prescribing. Standard sensitivity analyses (one-way and probabilistic) will be used to explore the robustness of conclusions to uncertainty in the underlying data.

Semi-structured telephone interviews will be conducted with the GPs and practice staff, including practice nurses and/or practice manager, from the intervention practices, after the intervention phase of the study Questions will focus on the acceptability and feasibility of the interventions, including the near patient testing (CRP study) in the practice and perceived impact on the management of ARIs. Interviews will be conducted after the full package has been implemented. All interviews will be recorded digitally and transcribed verbatim by the interviewer. Interview data will be analysed using inductive thematic analysis. Two researchers will independently code the interviews, compare their coding and discuss discrepancies. The final thematic framework will be constructed through an iterative process and tested by researchers with clinical expertise.

### Sub-studies

Specific studies are nested in the main study: (1) 1 point prevalence nose and throat swab study: asymptomatic carriage of bacterial upper respiratory pathogens; (2) feasibility of direct measures of resistance by nose/throat swabbing.

**Fig. 1:**
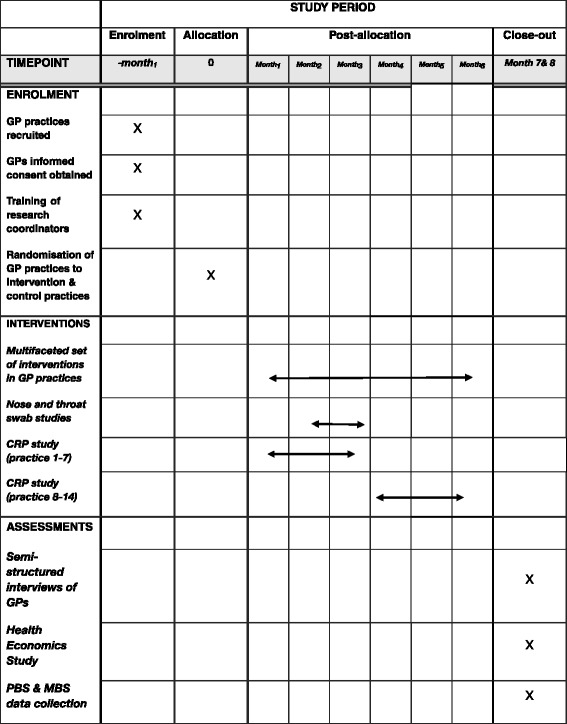
Schedule of enrolment, interventions, and assessments
  The ASPReN network will select 10 practices across Australia and each practice will identify five different patients who present with a non-infectious illness. The GPs will obtain consent and collect a throat and nose swab from each patient (for a total of 50 anterior nasal and 50 throat swabs). The swabs will be processed as described above.

1. *Point prevalence nose and throat swab study: asymptomatic carriage of bacterial upper respiratory pathogens*
  This pilot study seeks to assess the prevalence of common bacterial upper respiratory tract commensals and pathogens in the nose and throat swabs of general practice staff and patients attending the GP practice for consultation with non-infectious conditions; and the rate of antimicrobial resistance in organisms isolated. It is not clear whether general practice settings are more like hospital settings, where staff can have a higher risk of carriage of resistant organisms; or more like the community, with staff having similar carriage and resistance patterns to asymptomatic adults. Properly addressing this question will have implications for the sort of infection control practices necessary in General Practice settings.
  Anterior nasal and throat swabs will be taken from selectively recruited general practice staff and patients who have consented to be part of the sub-study. A total of 125 general practice staff and 125 patients across the practice sites will be included.
  Various appropriate bacterial media will be used to capture both potential pathogens and normal flora. The swabs will be cultured on selective media, such as mannitol salt agar and MacConkey agar to screen for *Staphylococcus aureus* and Gram-negative bacteria of respiratory pathogens such as *Klebsiella pneumoniae* and *Pseudomonas aeruginosa* [40]. Organisms isolated will be further evaluated for resistance to commonly used antibiotics with standard susceptibility testing [41]. Antibiotic resistant organisms will also undergo molecular characterisation to look for potential clonality and spread in the community [42].
2. *Rolling out community antibiotic resistance surveillance: ASPReN pilot*
  The study will assess the feasibility of surveillance of antibiotic resistance in primary care on a national basis utilising the Australian Sentinel Practice Network (ASPReN). ASPReN is a national network of GPs involved in surveillance activities including influenza. ASPReN will identify general practices in their network and provide simple instructions for taking and transporting throat and nasal swabs (Fig. 1). ASPReN will provide feedback about the feasibility of this surveillance activity.

### Ethics

Ethical approval has been obtained for the study from the University of Queensland (ref: 2015000988). In addition, administrative review has been obtained from Bond University and Queensland University of Technology ethics committees. The Department of Human Services has granted approval for consent to be obtained from the GPs to access their Medicare data (ref: MI4140).

### Trial status

This project has recruited the 28 GP practices and 110 GPs have consented to us accessing their Medicare data. The practices have been randomised to the intervention and control groups. Research coordinators have been appointed and trained in the interventions during the initiation phase of the study. The six month study period is currently underway.

## Discussion

This project will test evidence-based approaches designed to improve AMS in general practice settings in Australia. Each of the interventions has been demonstrated to be effective on its own at reducing antibiotic prescribing for ARIs elsewhere in the world [33], however, this is the first time that they have been combined into an integrated, multifaceted package, and delivered as an AMS toolkit. GPs’ antibiotic prescribing rates will be evaluated to estimate the effectiveness of the package. Its acceptability and feasibility from the GPs perspective will be evaluated, including obtaining feedback about how it may need to be further adapted for the Australian context. Currently there is limited information about the cost and cost effectiveness of AMS interventions, as most have not been evaluated or only looked at in a hospital context [43]. In undertaking an economic evaluation of our package to determine the costs of implementation and weigh these costs against the likely effect, we will provide important information on its relative efficiency. This kind of information is vital to decision makers seeking to design an efficient approach to AMS and maximise the benefits from recent investment in this area [17].

The sub-studies nested within the main trial will also add significantly to the knowledge base about antimicrobial resistance in the GP setting. There are very few data about community antibiotic resistance surveillance in non- infectious patients and staff. Colonisation with *Staphylococcus aureus* has been highly associated with the infections by *S. aureus* strains. In an Australian study, the rate of *S. aureus* colonisation amongst patients with blood stream infections by *S. aureus* was 58 % [44]. The majority of the *S. aureus* colonisation was in the nose (80 %). *S. aureus* colonisation in the throat was also common (24 %) in that study [44]. Approximately 15 % of *S. aureus* isolated from these patients were methicillin resistant *S. aureus* [44]. Thus far, there are no Australian data of *K. pneumoniae* and *P. aeruginosa* nasal carriage, the two important Gram-negative pathogens. *P. aeruginosa* intestinal carriage has been recently established [45].

The results on the effectiveness, cost-effectiveness, acceptability and feasibility of this package of interventions will support methods of operationalising an integrated AMS package in an Australian context in order to promote enhanced AMS initiatives.

## Abbreviations

AMS: Antimicrobial stewardship
ARI: Acute Respiratory Infections
ASPReN: Australian Sentinel Practice Network
ATC: Anatomical Therapeutic Chemical code
ChAP study: Changing the Antibiotic Prescribing of General Practice
CRP: C-reactive protein
GAPS: General Practitioner Antimicrobial Stewardship Programme Study
GPs: General practitioners
MBS: Medical Benefits Scheme
PBS: Pharmaceutical Benefits Scheme
